# Preparation and Characterization of Fenofibrate-Loaded Fibers Based on 2-Hydroxylpropyl-β-Cyclodextrin

**DOI:** 10.3390/polym17081037

**Published:** 2025-04-11

**Authors:** Enikő Bitay, Zoltán-István Szabó, Attila Levente Gergely

**Affiliations:** 1 Department of Mechanical Engineering, Faculty of Technical and Human Sciences, Sapientia Hungarian University of Transylvania, Calea Sighișoarei nr. 2, 540485 Târgu-Mureş, Romania; bitay.eniko@eme.ro; 2 Bánki Donát Faculty of Mechanical and Safety Engineering, Óbuda University, Bécsi Street 96/b, 1034 Budapest, Hungary; 3 Department of Drugs Industry and Pharmaceutical Management, George Emil Palade University of Medicine, Pharmacy, Science, and Technology of Targu Mures, Gh. Marinescu 38, 540485 Târgu-Mureş, Romania; 4 Sz-Imfidum Ltd., 525401 Lunga, Romania

**Keywords:** fenofibrate, cyclodextrin, fibers, solid dispersion, electrospinning

## Abstract

Fenofibrate is used to treat dyslipidemia, a health condition that could lead to cardiovascular diseases. Fenofibrate is classified as a class II drug by the Biopharmaceutical Classification System due to its high lipophilicity and low solubility in water. The purpose of this work was to enhance the dissolution characteristics of fenofibrate by incorporating it into 2-hydroxylpropyl-β-cyclodextrin fibers for the first time. Single-needle electrospinning was used to prepare the fenofibrate-loaded cyclodextrin fibers. The drug loading was optimized to fulfill the electrospinning conditions and was determined to be a 1:4 drug:cyclodextrin molar ratio. We found dimethylformamide a suitable solvent and were able to prepare bead-free fenofibrate-loaded 2-hydroxylpropyl-β-cyclodextrin fibers with an average diameter of 2.65 ± 0.82 μm. Drug loading was determined to be close to the theoretical value, 97.2%, with the aid of ultraviolet spectroscopy. Differential scanning calorimetry and Fourier transform infrared spectroscopy were used to track the crystalline to amorphous transition of fenofibrate through fiber formation. The dissolution results indicated a 60-fold increase in fenofibrate from the prepared fibers with respect to the micronized active ingredient.

## 1. Introduction

The introduction of high-throughput techniques in drug discovery has led to the development of numerous potent drugs; however, most of the newly developed drugs have several undesirable properties, such as high lipophilicity, high molecular weight, and low aqueous solubility [1]. An example of such a drug is fenofibrate (FEN), which is classified as a class II drug by the Biopharmaceutical Classification System due to its high lipophilicity and low solubility in water [2]. FEN is widely used to treat dyslipidemia, a health condition characterized by elevated levels of lipids and fatty substances (e.g., triglycerides and cholesterol) in the blood. If left untreated, dyslipidemia can lead to serious cardiovascular complications, such as heart attack or stroke, due to the formation of plaque in blood vessels [3,4,5].

A variety of methodologies have been employed [6] to enhance the solubility and bioavailability of FEN such as micronization [7,8], self-emulsifying drug delivery systems [9,10], nanoparticle formulations [11,12,13,14], insoluble drug delivery microparticulate formulation [15], and thin film freezing [16]. However, recently, fiber-based drug delivery systems have also attracted considerable attention in the context of improving solubility and bioavailability [17,18]. These systems inherently provide favorable physical properties such as nano-micrometers scale, high porosity, and high surface-to-volume ratio. Additionally, the amorphization of the drug during the fiber formation can positively influence the solubility of the drugs in aqueous conditions [19,20,21]. The most widely used technique for fiber production is electrospinning. The working principle of electrospinning has been discussed extensively in the literature [22,23,24,25,26]. The process involves dissolving the fiber-forming polymer in a suitable solvent, followed by the application of a high electric field on a droplet of the solution that forms at the end of a capillary. Under optimal conditions, the so-called Taylor cone [27,28] (solution droplet with the distorted shape) is generated and a polymer solution jet forms. This jet travels from the droplet to the grounded collector due to the applied electric field. It is noteworthy that during this process the solvent undergoes significant evaporation, leading to substantial jet elongation due to the bending instabilities. The outcome of this process is the deposition of dry nanometer-scale fibers on the collector.

Fiber-based FEN-loaded amorphous solid dispersion was prepared by Sipos et al. [29] using electrospinning with the objective of enhancing the solubility of the active version. Polyvinyl pyrrolidone (PVP) was utilized as the carrier polymer, while ethanol (EtOH) was employed as the solvent. The authors encountered challenges during the dissolution of FEN in the PVP/EtOH solution. To address this challenge, a series of surface-active agents were tested and polysorbate 80 (Tween 80) was selected to prepare the drug-loaded solution. The authors prepared bead-free randomly oriented FEN-loaded PVP fibers with an average fiber diameter of d = 1.10 ± 0.23 μm. The FEN loading of the fiber mat was 6.5 ± 0.09 *w*/*w*. Fourier-transformed infrared spectroscopy and positron annihilation lifetime spectroscopy were employed to determine the amorphous state of FEN in the PVP fibers. The dissolution studies indicated a 40-fold increase in the dissolution amount from the FEN-loaded fibers compared to the micronized crystalline FEN. We have previously [30] used corona electrospinning, a scale-up technique, to prepare FEN-loaded PVP fibers. The drug loading was 5.31 ± 0.02 *w*/*w*, whereas the fiber diameters ranged from 1.4 to 4 μm. By comparing the dissolution studies of FEN-loaded PVP fibers prepared by single needle and corona (needleless) electrospinning, one could conclude that both displayed similar characteristics. These were the first examples of the preparation of FEN-loaded polymer fiber-based amorphous solid dispersions.

A variety of polymeric materials have been successfully utilized to prepare a fiber-based drug delivery system with electrospinning, such as PVP [31], polyvinyl alcohol (PVA) [32], gelatin [33,34], chitosan [35,36,37], and others. Recently, cyclodextrins (CDs) and their derivatives have gained substantial attention in replacing partially [38,39] or completely [40,41] polymeric matrix materials. The appeal of CDs in the domain of drug delivery applications stems from their nontoxic nature, low immunogenicity, remarkable water solubility, and extensive modification potential [42]. CDs are cyclic oligosaccharides comprising 6, 7, or 8 glucose units, thus composing the α-, β-, and γ-CDs, respectively. CDs are characterized by a toroidal three-dimensional shape with a hydrophilic outer shell and a hydrophobic inner cavity, in which lipophilic drugs can enter to form inclusion complexes, thus improving their solubility [42]. The water solubility and complexation properties of the native CDs can be improved by modifications; thus, substituted CDs are frequently applied for drug delivery applications [43]. Among these, 2-hydroxypropyl-β-cyclodextrins (HP-β-CDs) emerged as the most widely applied derivative for polymer-free nanofibrous formulation [44,45,46].

In recent years, CDs have garnered attention in the field of drug delivery applications due to their advantageous properties. Several studies have emerged on the use of CDs in conjunction with polymeric materials to enhance the solubility of poorly soluble drugs by preparing nanofibers [45]. However, it has been demonstrated that fibers can be produced from highly concentrated CD solutions without the use of a carrying polymeric material [47,48]. The use of CD fibers has been demonstrated to enhance the solubility and bioavailability of several poorly soluble drugs, such as sulfisoxazole [49], diclofenac sodium [50], voriconazole [51], and metronidazole [52] amongst others [44].

The objective of this study was to develop HP-β-CD-based fibers loaded with FEN for the first time to enhance the solubility of FEN. The optimization of electrospinning parameters was also targeted to attain a stable and high-yield production of FEN-loaded fiber mats. The physico-chemical changes occurring during the electrospinning process were monitored using DSC and FT-IR studies, while disintegration and dissolution studies were conducted to assess the performance of the prepared fibrous material.

## 2. Materials and Methods

### 2.1. Materials

Fenofibrate (FEN, courtesy of a local pharmaceutical company, in Targu Mures, Romanian), dimethyl formamide (DMF, reagent grade Sigma Aldrich, Darmstadt, Germany), and 2-hydroxypropyl-β-cyclodextrin (HP-β-CD, DS~4.5, Apollo Scientific, Stockport, UK) were used as received without further purification. Sodium dihydrogen phosphate (NaH_2_PO_4_), potassium dihydrogen phosphate (KH_2_PO_4_), sodium chloride (NaCl), orto-phosphoric acid (85%, *w*/*w*), and acetonitrile (ACN) were obtained from Merck (Darmstadt, Germany). Ultrapure water was prepared with a Barnstead Nanopure Diamond water purification system (Boston, MA, USA).

### 2.2. Fiber Preparation by Electrospinning

To determine stable electrospinning conditions, different HP-β-CD:FEN molar ratios were tested. The HP-β-CD concentration was maintained at 140% *w*/*v* during the preliminary experiments, as this concentration has previously yielded good results. The solutions were prepared by dissolving the required amount of HP-β-CD in DMF with the aid of stirring, at 500 rpm, with a magnetic stirrer (Phoenix Instruments RSM-10HS magnetic stirrer (Garbsen, Germany)) for approximately 30 min at 65 °C, resulting in a viscous clear solution. FEN stock solution (concentration: approximately 361 mg/mL FEN) was also prepared by dissolving the appropriate amount of the active substance in DMF by magnetic stirring at 500 rpm for approximately 10 min at room temperature, resulting in a clear solution. Subsequently, the predetermined amount of FEN solution was added in small aliquots to the HP-β-CD solution at room temperature while stirring at 500 rpm. This resulted in a clear viscous solution after approximately 10 min. Three solutions were prepared in 1:1, 2:1, and 4:1 HP-β-CD:FEN molar ratios.

Electrospinning was carried out on a custom-made electrospinning setup. The positive potential of the high-voltage power supply (Gamma High Voltage Research, Ormond Beach, FL, USA) was connected to a needle while the collector was grounded. The constant volumetric flow rate was provided by a syringe pump (KD Scientific, Holliston, MA, USA). The FEN-containing solution was loaded into a 5 mL syringe that was connected to an 18 G needle with PTFE tubing. Stable electrospinning conditions were established to be a 20 kV applied voltage, 240 mm needle-to-collector distance, and volumetric flow rate of 4 mL/h. The electrospinning process was performed at ~22 °C and ~35% RH.

### 2.3. Scanning Electron Microscopy (SEM)

Morphological characterization of the prepared fiber mats was performed on a JSM-5200 scanning electron microscope (JEOL, Tokyo, Japan) on neat, not sputter coated, samples using a 15 kV accelerating voltage. The average fiber diameter was determined with ImageJ (version 1.52a, National Institutes of Health, Bethesda, MD, USA) open-access software using 100 measurements from different parts of the samples. The average fiber diameter and standard deviation were subsequently calculated using the equations of the normal distribution function.

### 2.4. Viscosity Measurement

The dynamic viscosity of the solutions was measured with an IKA ROTAVISC lo-vi viscometer (IKA, Königswinter, Germany) at 22.5 ± 0.5 °C. The measurements were performed using the SP-2 spindle at 2 rpm. Based on the dimensions of the spindle and the container, the calculated shear rate was 15.36 1/s. The dynamic viscosity of the solutions was calculated based on 5 measurements which were recorded 1 min after starting the rotation of the spindle.

### 2.5. Conductivity Measurements

The electric conductivity properties of the solutions containing only the HP-β-CD and both HP-β-CD and FEN were investigated using a conductivity meter from VWR, HCO 304 at 22 °C. Conductivity measurements were performed three times.

### 2.6. Fourier Transform Infrared (FT-IR) Spectroscopy

FT-IR measurements were performed on a Bruker Tensor 27 IR spectrometer (Bruker Optics, Ettlingen, Germany), controlled by the Opus software (version 7.2). The IR spectra of the individual components (FEN, HP-β-CD), their physical mixture, and the microfibrous sample were collected in transmittance mode over the 400–4000 cm^−1^ wavenumber range. For each sample, 16 scans were performed at a resolution of 2 cm^−1^.

### 2.7. Drug Content Determination of the FEN-Loaded Fibers

**Figure 1:**
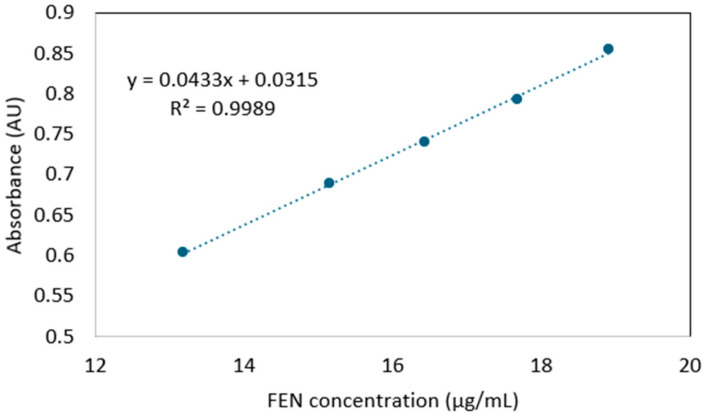
Calibration curve used for the determination of the FEN content of nanofibers.

Three samples of approx. 13 mg were taken randomly from the FEN-loaded fiber mats and individually dissolved in 50 mL methanol by ultrasonication for 2 min. Overall, 1 mL of the obtained solution was further diluted to 10 mL with methanol and analyzed in a 10 mm quartz cuvette by UV spectroscopy, using a Shimadzu UV-1601PC spectrophotometer. The spectra were recorded in the 250–350 nm range. In order to determine the FEN content, a calibration curve was constructed in five points in the concentration range of approximately 13–19 μg/mL FEN in methanol (Figure 1).

The loading efficiency was determined using the following equation given below:

(1) $$\mathrm{Loading}\;\mathrm{efficiency}\;\left(\%\right)=\frac{C_{p}}{C_{t}}\times \;100$$

where *C_p_* is the practically obtained (measured) FEN concentration and *C_t_* is the theoretical FEN content of the dry fibers.

### 2.8. Differential Scanning Calorimetry (DSC)

Thermoanalytical studies were performed using a Shimadzu DSC-60 (Shimadzu, Tokyo, Japan) differential scanning calorimeter. Samples with a mass ranging between 3 and 9 mg were placed into aluminum pans. An empty aluminum pan was used as a reference for the measurements. The DSC measurement consisted of a single heat cycle in the temperature range of 30–150 °C, using a 5 °C/min heat rate under air atmosphere.

### 2.9. In Vitro Dissolution Tests

The dissolution tests were carried out using a custom-built apparatus as previously detailed in our publications [53,54]. Glass tubes measuring 11.5 cm × 2.7 cm (height × ID) were immersed in a water bath that was maintained at 37 ± 1 °C. This was accomplished using an Erweka ET 1500I immersion thermostat (Erweka GmbH, Heusenstamm, Germany). The dissolution media comprised 50 mL of artificial saliva (2.38 g/L NaH_2_PO_4_, 0.19 g/L KH_2_PO_4_, 8 g/L NaCl in water) was used. Stirring at 200 rpm was performed employing a Phoenix Instruments RSM-10HS magnetic stirrer (Garbsen, Germany), using Teflon-coated stir bars. At predetermined time points (1 min, 3 min, 5 min, 10 min, 15 min, 20 min, and 30 min), 2 mL samples were withdrawn and filtered through a 0.45 μm Chromafil Xtra PVDF filters (Macherey-Nagel, Düren, Germany) and replaced with fresh preheated dissolution media.

The quantification of the dissolved active substance was performed using an isocratic HPLC method using a Thermo Finnigan Surveyor HPLC, consisting of a quaternary pump, autosampler, column thermostat, and a diode-array detector (DAD). The analytical column used was a Purospher STAR RP-18e (150 × 4.6 mm, 5 μm), thermostated at 50 °C, while the mobile phase consisted of 10% 0.1% (*v*/*v*) orto-phosphoric acid in water (A) and 90% acetonitrile (B). The flow rate was 1.5 mL/min. Under these conditions, the retention time of FEN was approximately 2.3 min. UV detection was performed at 286 nm.

## 3. Results

### 3.1. Fiber Morphology

**Figure 2:**
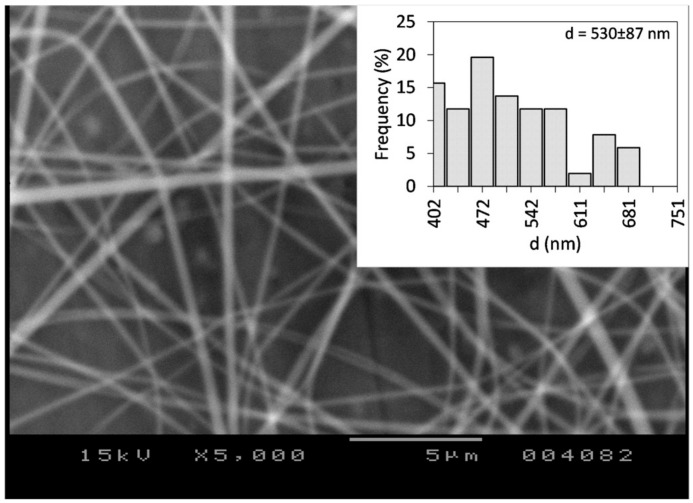
SEM image of neat HP-β-CD fibers from water (c = 190% *w*/*v*).

It has been reported that CDs have the capacity to increase the solubility of drugs that are inherently poorly water-soluble. Consequently, a solution of CD and the drug can be prepared using only water as the solvent [52,55]. Initially, the electrospinnability of the HP-β-CD in water was investigated. Thus, neat HP-β-CD solution was prepared in water at different concentrations. The results indicated stable electrospinning conditions at 190% *w*/*v* concentration, V = 18 kV applied voltage, D = 150 mm needle-to-collector distance, and F = 1 mL/h volumetric flow rate. The average fiber diameter of the prepared fibers was determined to be d = 530 ± 87 nm. The optimal conditions resulted in a stable electrospinning process, as also indicated by the low standard deviation value. An SEM image and a histogram of the prepared fiber mat is shown in Figure 2. The fibers exhibited random orientation and were devoid of beads featuring smooth surfaces.

After a stable production process was attained for neat HP-β-CD solution, the incorporation of FEN into the spinning solution was attempted. The FEN content was systematically increased; however, upon the attempt to mix the FEN into the HP-β-CD/water solution, a hazy suspension formed even after 24 h of mixing. Despite this, an electrospinning process was attempted but it proved unsuccessful in producing fibers.

**Figure 3:**
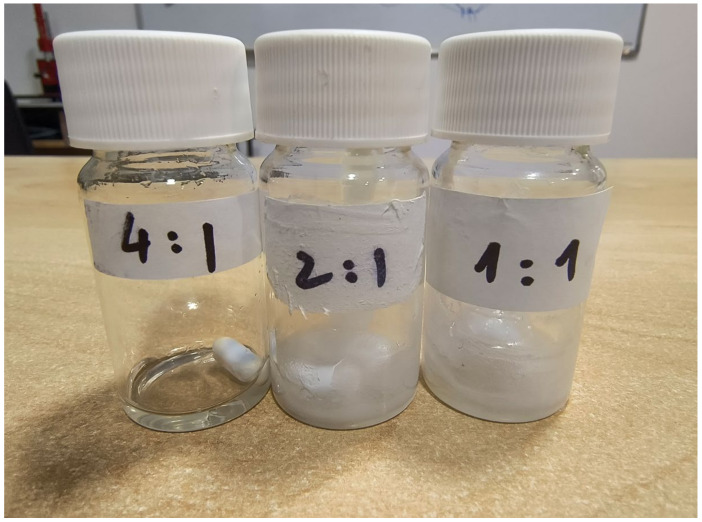
Physical appearance of solution containing different HP-β-CD:FEN ratios in DMF.

**Figure 4:**
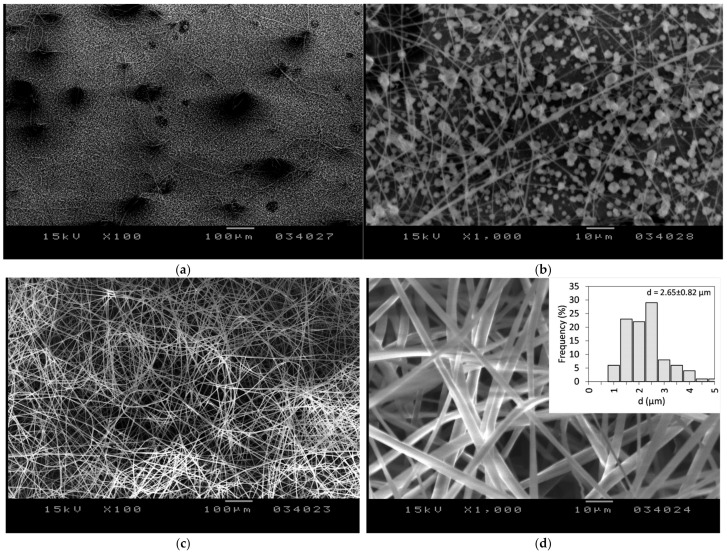
SEM images of a 2:1 CD:FEN molar ration (**a**) 100×; (**b**) 1000× and 4:1 CD:FEN molar ratio (**c**) 100×, (**d**) 1000×.

Subsequently, DMF was examined as a potential solvent given its ability to dissolve both FEN and CDs [47]. It was determined that stable electrospinning conditions require a concentration of 140% *w*/*v* HP-β-CD, which aligns closely with previously reported literature data [47]. Furthermore, three different molar ratios of 1:1, 2:1, and 4:1 HP-β-CD:FEN were investigated. In the case of a 1:1 molar ratio of HP-β-CD:FEN, a hazy very viscous solution was obtained (Figure 3) and we were unable to produce fibers. A similar hazy solution was obtained using the 2:1 HP-β-CD:FEN ratio; however, in this case, the electrospinning process resulted in beaded fibers (Figure 4b) with solution droplets present on the sample (Figure 4a).

The conductivity of HP-β-CD in DMF and a 4:1 HP-β-CD:FEN molar ratio in DMF solutions were investigated, finding it to be 1 μS/cm in both cases. Consequently, it can be deduced that the incorporation of FEN did not alter the conductivity of the solutions as was expected since FEN is an electrically neutral molecule. The viscosity of the prepared solutions was also determined, as there is a direct correlation between the solution viscosity and the resulting fiber diameter, meaning an increase in spinning solution viscosity results in thicker fibers. The dynamic viscosity of the HP-β-CD solution in DMF at 140 *w*/*v* concentration was measured to be 6809 ± 10 mPs·s. Upon the introduction of FEN (4:1 molar ratio HP-β-CD:FEN), the viscosity increased to 11,729 ± 21 mPa·s. Both conductivity and viscosity of a solution are fundamental properties that influence the electrospinning ability. The introduction of FEN to the HP-β-CD solution did not result in a change in conductivity; however, it led to a substantial increase in viscosity, which consequently increased the diameter of the produced fibers.

A transparent viscous solution (Figure 3) was obtained when a 4:1 HP-β-CD:FEN ratio was employed to prepare the solution for electrospinning. Using this solution, stable electrospinning condition was attained, with the following parameters: V = 20 kV applied voltage, D = 240 mm needle-to-collector distance, and F = 4 mL/h volumetric flow rate. Fibers were collected for ~15 min. The SEM images of the produced FEN-loaded HP-β-CD fibers are presented in Figure 4c,d. The resulting fibers were found to be devoid of beads, exhibited random orientation, and were characterized by smooth surfaces. The average fiber diameter was determined to be d = 2.65 ± 0.82 μm. In comparison to the fiber diameter of HP-β-CD from water solution (d = 530 ± 87 nm), it can be concluded that the diameter is larger, which is partly due to the higher viscosity of the prepared solution. The results align with the trend reported in the literature, which suggests that when DMF is as a solvent instead of water, the average diameter of the resulting fibers increases [47].

### 3.2. Determination of the FEN Content of the Nanofibers

**Figure 5:**
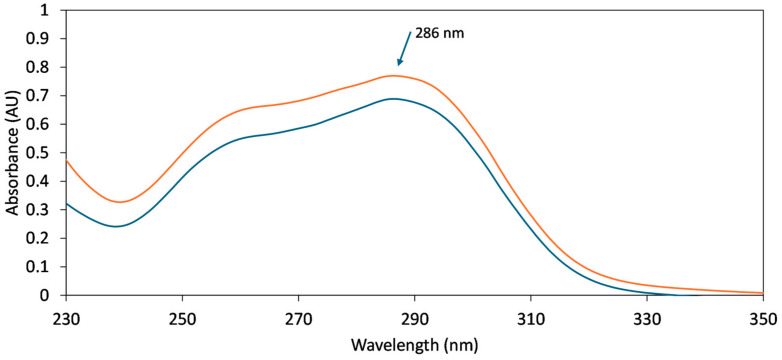
Overlaid representative UV spectra obtained for FEN standard solution (blue) and FEN-loaded nanofiber sample solution (orange).

UV spectroscopic studies were conducted to demonstrate the incorporation of the active ingredient into the fibrous mats and to quantify its concentration. Representative overlaid UV spectra are displayed in Figure 5, indicating that the same absorbance maxima at 286 nm can be observed in both cases: standard solution and the sample solution prepared from the FEN loaded HP-β-CD fibers. Furthermore, the quantitative results indicated loading efficiency values close to 100% (average loading efficiency = 97.2%), with a low standard deviation, indicating a homogenous dispersion of the active substance in the fibers (Table 1).

### 3.3. Thermoanalytical Studies

**Figure 6:**
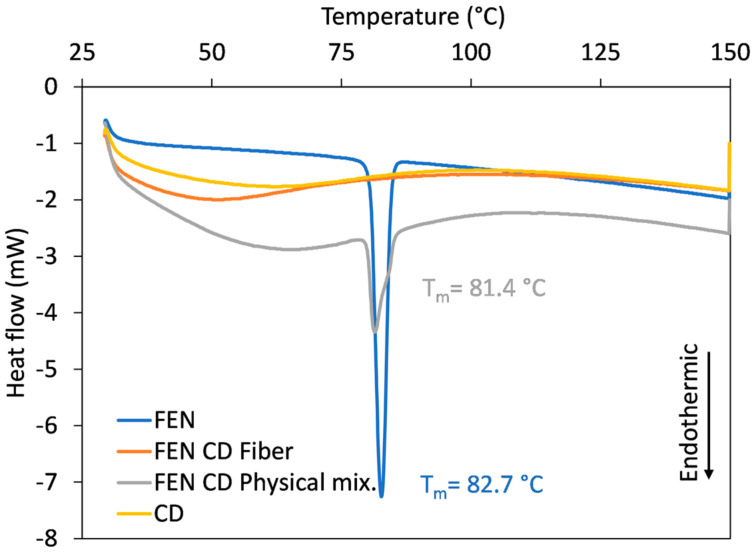
Overlaid DSC thermograms of the individual components, the physical mixture, and the obtained nanofibrous mats.

To track the physical changes that occur during the electrospinning process, thermoanalytical studies using DSC were performed. The DSC thermograms of the individual components (FEN and HP-β-CD), the physical mixture of the components, and the obtained nanofibers were recorded and can be observed in Figure 6. The physical mixture of FEN and HP-β-CD was prepared in such a way that the FEN content matched with the FEN content of the FEN-loaded HP-β-CD fibers.

The thermogram of the amorphous HP-β-CD showed only a wide endothermic event between 30 and 75 °C due to the dehydration of the HP-β-CD. The DSC curve of FEN exhibited a single endothermic event, corresponding to the melting of the crystalline active substance. The obtained endothermic peak at 82.7 °C (onset temperature at 80.57 °C and endset temperature of 84.58 °C) is consistent with the previously reported data. Both thermal events can be observed on the DSC curve of the physical mixture: the dehydration of the amorphous HP-β-CD and the superpositioning of melting event of the dispersed crystalline active substance. The melting of the active ingredient, FEN, happened at 81.40 °C (onset temperature at 79.90 °C and endset temperature of 85.38 °C), indicating some degree of interaction between the FEN and HP-β-CD. The peak broadening noticed at the physical mixture compared to the neat FEN also supports this hypothesis. Furthermore, the interaction between the FEN and HP-β-CD could decrease crystallinity [56]. The thermogram of FEN-loaded HP-β-CD fibers contain the dehydration peak of HP-β-CD between 30 and 75 °C, and the endothermic peak corresponding to the melting temperature of FEN disappears. This result could indicate a potential crystalline to amorphous transition of FEN when incorporated in the HP-β-CD fibers through electrospinning.

### 3.4. Fourier Transform Infrared (FT-IR) Spectroscopy Studies

**Figure 7:**
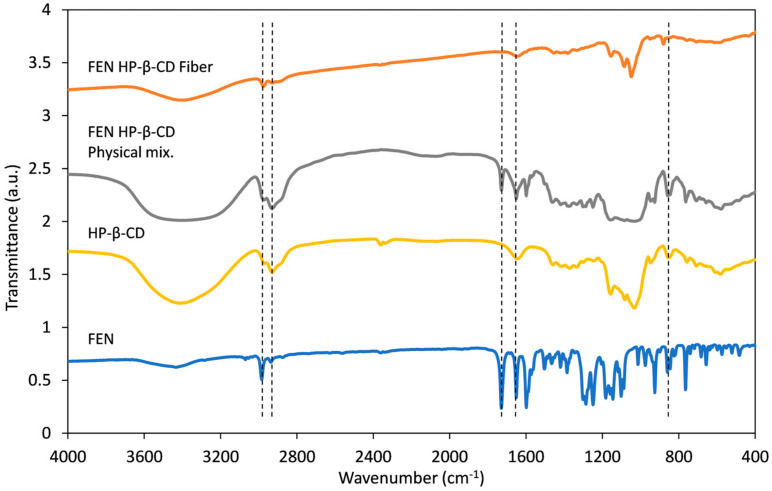
Overlaid FT-IR spectra of the individual components, the physical mixture, and the obtained nanofibrous mats.

The FT-IR analysis was performed to investigate the interaction between the FEN and HP-β-CD. The FT-IR spectra of FEN, HP-β-CD, their physical mixture, and the FEN-loaded HP-β-CD fibers are shown in Figure 7. The spectra of the individual samples were shifted vertically for better representation. Characteristic peaks are presented with vertical dashed lines.

The FT-IR spectrum of HP-β-CD contains bands corresponding to oligosaccharides, most notably at 3400 cm^−1^ (O-H stretching vibration), 2930 cm^−1^ (C-H stretching vibration), 1640 cm^−1^ (O-H bending vibration), and 1155 cm^−1^ (C-O vibration). The methyl group of the hydroxypropyl moiety is identified at 2960 cm^−1^ with the anti-symmetric vibration. The glucopyranosel units are connected by an α-type glycosidic bond indicated by the characteristic peak at 855 cm^−1^ [57]. The spectrum of FEN presents characteristic peaks at 1729.53 cm^−1^ from the C=O vibration absorption, 1651.08 cm^−1^ from the methyl ester carbonyl group C=O bond, and 2983 cm^−1^ attributed to the C-H stretching [58]. The spectrum of the physical mixture of FEN and HP-β-CD is basically an overlay of the two individual spectra. The C=O stretching of FEN at 1729.53 cm^−1^ and 1651.08 cm^−1^ shifted to 1731 cm^−1^ and 1649 cm^−1^ and broadened in the case of the physical mixture. The shift and the peak broadening at 3400 cm^−1^ indicate some degree of interaction between the amorphous FEN and the HP-β-CD [58,59]. This result corresponds well with the DSC results presented in Figure 6. In the case of FEN-loaded HP-β-CD fibers, the characteristic peaks of HP-β-CD can be observed; however, the intensity of the characteristic peaks of FEN decreased substantially. This indicates that there is an interaction between FEN and HP-β-CD [58].

### 3.5. Disintegration Test

**Figure 8:**
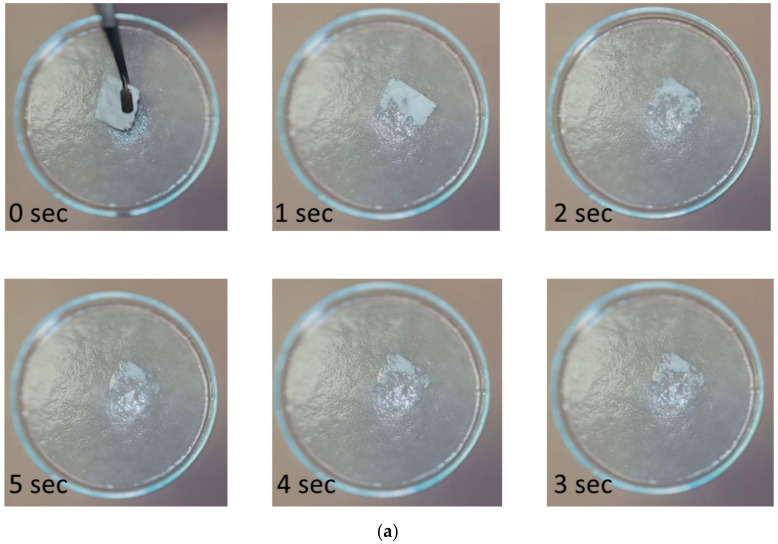

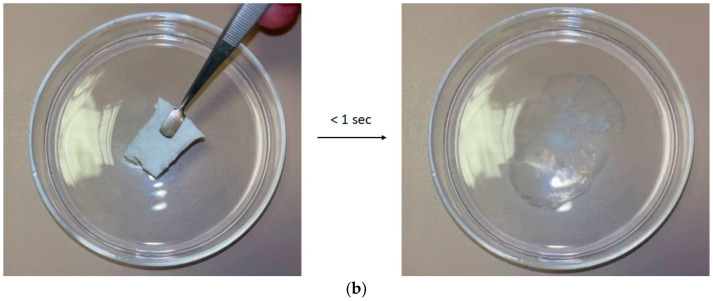
Disintegration studies (**a**) in a simulated moist tongue environment and (**b**) in water.

Disintegration tests were conducted both in a simulated moist tongue environment, using a filter paper wetted with artificial saliva, as well as in water. Upon contact with the wet filter paper (Figure 8a), the fibrous mats displayed spontaneous disintegration and liberation of the active substance. Similar characteristic was observed when the FEN-loaded HP-β-CD fiber mat was immersed in water (Figure 8b), where the disintegration occurred within 1 s. The rapid disintegration can be attributed to the combined effect of the high porosity and high surface-to-volume ratio of the fiber mat, the amorphous nature, and the high solubility of the CD derivative.

### 3.6. In Vitro Release Studies

**Figure 9:**
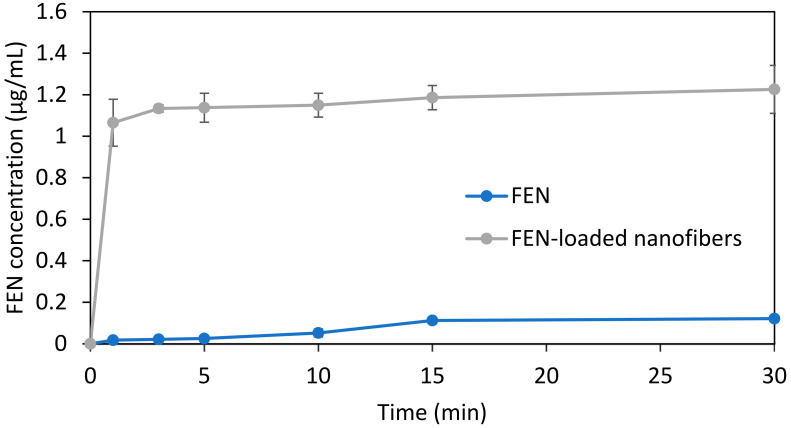
Comparative dissolution profiles of neat FEN (blue) and FEN-loaded CD nanofibers (gray).

To assess the release of the active ingredient, a small-volume in vitro dissolution test was conducted using artificial saliva. The test was performed on both the active substance and the drug-loaded HP-β-CD fibers. The results of these tests are summarized in Figure 9. The active substance exhibited an exceptionally low solubility, with a negligible increase in concentration over time to 0.12 μg/mL. As demonstrated, the microfibrous formulation undergoes immediate disintegration upon contact with the aqueous environment and displays a burst release of the active substance, which rapidly plateaus from the first time points of 1.07 μg/mL to 1.22 μg/mL after 30 min. It is interesting to observe that at 1 min, FEN release is over 60 times higher from the microfibrous formulation, compared to the micronized active ingredient.

The results of our study are well aligned with those reported in the literature concerning the use of CD derivatives in the preparation of fiber-based fast-dissolving formulations. Celebioglu et al. used HP-β-CD to enhance the solubility of metronidazole [52]. The authors used 1:1 and 1:2 drug:HP-β-CD ratios to prepare drug-loaded fibers. The dissolution studies showed that at the initial data point (30 s), the maximum amount of drug was released from the prepared nanofibers for both molar ratios; however, there was a slow dissolution of the micronized drug. Based on the dissolution results, the authors achieved a ~5- and ~6-fold increase for the 1:1 and 1:2 metronidazole:HP-β-CD molar ratios, respectively. However, after 30 min, the dissolution of the micronized metronidazole reached levels comparable to those of the 1:1 ratio.

A comparison of the dissolution characteristics of FEN from polymer-based fibers revealed similar trends to our results. The dissolution of FEN was found to be enhanced by a factor of 40 from FEN-loaded PVP fibers prepared by single-needle electrospinning and needleless corona electrospinning [29,30]. Additionally, it can also be concluded that fiber-based dissolution performance is on par with other dissolution enhancement methods, for example, co-grinding and kneading. Kondoros et al. reported solubility enhancement of FEN with the co-grinding method [8]. The authors found that the solvent-free co-grinding method produced a FEN and heptakis-(2,6-di-O-methyl)-β-CD formulation that performed better with regard to FEN dissolution when compared to the kneaded formulation. The authors also found that FEN dissolution from the co-grinded formulation increased the solubility of FEN ~25-fold compared to the micronized FEN after 30 min. Consequently, based on our findings and the literature data, we can hypothesize that CD derivatives are promising candidates for FEN dissolution enhancement.

## 4. Conclusions

In conclusion, electrospinning was successfully employed to prepare FEN-loaded HP-β-CD fibers. The results suggest that HP-β-CD fibers can be prepared using both water and DMF; however, the incorporation of FEN into HP-β-CD fibers is only possible when DMF is used as solvent. The optimal molar ratio of FEN and HP-β-CD was determined to be 1:4, whereas the HP-β-CD concertation in DMF was found to be 140% w/v, which resulted in a solution suitable for electrospinning. The preparation of bead-free randomly oriented smooth surfaced fibers was achieved under the optimal electrospinning conditions of 20 kV applied voltage, 4 mL/h volumetric flow rate, and 240 mm needle-to-collector distance while using an 18 G needle as the capillary. The average fiber diameter was measured to be 2.65 ± 0.82 μm. The dissolution results indicate that the solubility of FEN was increased by 60-fold for the FEN-loaded HP-β-CD fibers, when compared to the micronized drug. The findings of this study are consistent with the reported literature data. Furthermore, the study demonstrates a higher solubility increase for FEN, indicating that the use of CD-based fibers emerges as a promising alternative to enhance the solubility of poorly water-soluble drugs.

## Figures and Tables

**Table 1:** Results of the determination of the FEN content of the fibers.

Sample No.	Measured FEN Content (%)	Theoretical FEN Content (%)	Loading Efficiency (%)	Average Loading Efficiency (%)	Standard Deviation
1	5.82	6.04	96.3	97.2	1.10
2	5.85		96.8		
3	5.95		98.4		

## Data Availability

The original contributions presented in this study are included in the article. Further inquiries can be directed to the corresponding author.
